# Impact of lexical and sentiment factors on the popularity of scientific papers

**DOI:** 10.1098/rsos.160140

**Published:** 2016-06-22

**Authors:** Julian Sienkiewicz, Eduardo G. Altmann

**Affiliations:** Max Planck Institute for the Physics of Complex Systems, 01187 Dresden, Germany

**Keywords:** citation analysis, sentiment analysis, quantile regression

## Abstract

We investigate how textual properties of scientific papers relate to the number of citations they receive. Our main finding is that correlations are nonlinear and affect differently the most cited and typical papers. For instance, we find that, in most journals, short titles correlate positively with citations only for the most cited papers, whereas for typical papers, the correlation is usually negative. Our analysis of six different factors, calculated both at the title and abstract level of 4.3 million papers in over 1500 journals, reveals the number of authors, and the length and complexity of the abstract, as having the strongest (positive) influence on the number of citations.

## 1. Introduction

The number of citations an article receives can be considered a proxy for the attention or popularity the article achieved in the scientific community. Citations play a crucial role both in the evolution of science [1–5] as well as in the bibliometric evaluation of scientists and institutions; in that case the number of citations is often tacitly taken as a measure of quality. Understanding which factors in a paper contribute or correlate with citations has been the subject of a number of investigations (see [6–8] for reviews). Diversity in the affiliation of authors, multinationality, multidisciplinarity, and number of references, figures or tables have all been identified as factors that positively correlate with citations.

Here, we perform a more systematic investigation of how different textual properties of scientific papers affect the number of citations they acquire (see §4.1 for data description). A classical result, which motivates our more general analysis, is the negative correlation between title length and citations (i.e. shorter titles, more citations) [9–12]. In our analysis, we consider additionally the complexity and the sentiment of the text both in the title and the abstract (table 1). Lexical complexity is usually considered as proportional to the effort needed (by non-experts) to understand the texts. We use three measures of text complexity (table 1) that take into account the number of different words in the text (normalized by the length) and the length of these words in syllables (see §4.2 for details). In several previous studies, authors used the concept of the sentiment analysis (i.e. emotional content) of the examined text/messages. In general, psychologists are able to specify several dimensions of emotions, reaching as far as 12 [14]. However, two of them—*valence* and *arousal*—are probably the best recognized and the most frequently used. Valence reflects the emotional sign of the message (negative, neutral, positive), whereas arousal is used to describe the level of activation (low, medium, high). Pairs of valence and arousal can indicate the specific emotion type [15], e.g. fear (negative and aroused), sad (negative and not aroused), etc.; however, they can also be used as independent variables. For example, valence as a standalone dimension has successfully been used to detect collective states of online users [16], to indicate the end of online discussions [17] or to predict the dynamics of Twitter users during Olympic Games in London [18]. Lately, this kind of analysis has also been introduced to judge upon the role of negative citations [19], citation bias [20] and to check what boosts the diffusion of scientific content [21]. Here, we quantify arousal and valence through dictionary classifier, see §4.3.

**Table 1.** List of textual factors whose relation to citations we investigate in our paper. Whenever possible, factors are obtained on the title and abstract of a paper.See §§4.2 and 4.3 for exact definitions. Additionally, we consider the number of authors (motivated by previous studies, e.g. [7,13]).

property	title	abstract
*length*	number of characters	number of words
*complexity*	—	Gunning fog index *F*
	*z*-index	*z*-index
	Herdan's *C*	Herdan's *C*
*sentiment*	valence	valence
	arousal	arousal
*number of authors*		

## 2. Results

**Figure 1.**
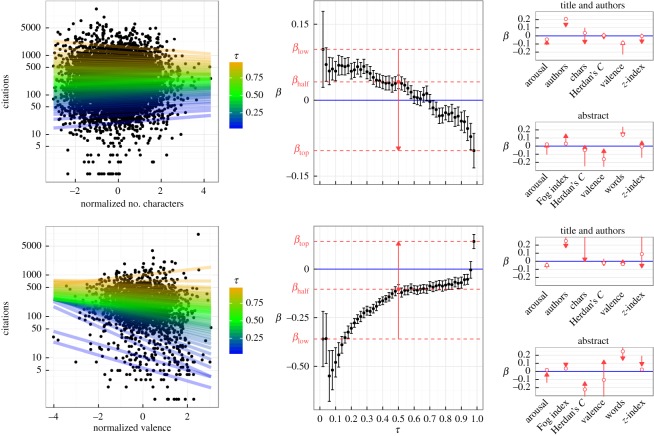
Relation between different factors and the number of citations in two journals: *Science* (top) and *Nature Genetics* (bottom). Left-side plots: each black dot corresponds to one paper, and lines show quantile regression (QR) results for colour-coded quantiles *τ*={0.02,0.04,…,0.98}. Middle panels: *β* coefficients (slopes of QR in the left panel) as a function of quantile *τ*. The red arrows (*summary pointers*) show *β*_low_≡*β*(*τ*=0.02), *β*_half_≡*β*(*τ*=0.5) and *β*_top_≡*β*(*τ*=0.98), as, respectively, the nock, a circle on the shaft, and the head of the arrow. Right panels: summary pointers for all factors.

We are interested in quantifying the relationship between *X*—a real number that quantifies for each paper one of the textual factors listed in table 1—and the logarithm of the number of citations Y≡ln⁡(citations+1). We standardize *X* in order to be able to compare the different factors (see §4.4), and we use the citations provided by Web of Science at the end of 2014 for papers published in 1995–2004.^1^ Exemplary results of the *X* versus *Y* relationship for two factors in two journals are shown in the left part of figure 1. The broad scattering of the points shows that visual inspection fails even to detect whether the relation between *X* and *Y* is positive or negative. The simplest (and widely used) approach is to perform an ordinary (least square) linear regression *Y* =*α*^†^+*β*^†^*X*, where *β* is related to the Pearson correlation coefficient *r* as *β*=*rσ*_*Y*_/*σ*_*X*_ (in fact, owing to standardization of variable *X*, in our case, *β*^†^ is simply *cov*_*XY*_). For the data in figure 1, this yields: *β*^†^=0.020±0.011 with *p*>0.05 for title length in *Science* and *β*^†^=−0.21±0.03 with *p*<0.001 for valence in *Nature Genetics*. In other words, the second example shows a negative correlation between valence and citations, whereas the first shows no clear correlation between the number of characters and citations (we cannot reject the null hypothesis of lack of linear dependence at 5% significance level). We note that the analysis of reference [12], which identified a negative correlation between title length and citation, was restricted only to the most cited papers. This difference in the conclusion regarding the role of title length and the large variability shown in the data motivates us to go beyond the above-described computation of linear correlations, which relies on the (homoscedasticity) assumption of uniform errors in the *whole* dataset.

### 2.1 Quantile regression

Quantile regression [22] is a method that tracks the relation between variables for different *parts* of the dataset. The simple question it addresses is: what are the coefficients *α* and *β* of a linear relation *Y* =*α*(*τ*)+*β*(*τ*)*X* that divides the dataset, so that a fraction *τ* of points lies below the line and the remaining part (1−*τ*) above it (a precise formulation of quantile regression (QR) is shown in §4.5). We thus obtain a sequence of values *β*(*τ*) that can be thought of as the quantification of the relation between *X* and *Y* at the *τ* quantile. The QR is widely used in different fields [23] and has lately been applied to predict future paper citation based on their previous history, i.e. early citations as well as on the Impact Factor (IF) [24].

The results in the centre panels of figure 1 show a clear *τ* dependence of *β*, a signature of the nonlinearity of correlations. For instance, the top panel shows that for low values of *τ* there is a positive correlation between number of characters in the title and citations, whereas for high *τ*, the correlation is reversed. This shows the limitations of the popularized message [25,26] following reference [12] that shorter titles lead to more citation. This only holds if you know in advance that your paper will be among the top-cited papers (longer titles seem to be better, e.g. in order to avoid being among the least cited papers). Similar observations (with the opposite trend) are observed in the bottom panel for valence—the emotional polarity—contained in the abstract of *Nature Genetics* articles. These examples show that even simple textual variables can have a mixed relation to the number of citations acquired by the papers of a given journal. We repeated the QR analysis for all factors in more than 1500 journals.^2^ In our discussion of our different findings below, we focus on three characteristic values of *β* which represent the low-cited (*β*_low_≡*β*(*τ*=0.02)), typical (*β*_half_≡*β*(*τ*=0.5)) and top-cited (*β*_top_≡*β*(*τ*=0.98)) papers (graphically represented in the central and right panels of figure 1 by a *summary pointer*, i.e. a red arrow with a circle).

### 2.2 Strength of factors

**Figure 2.**
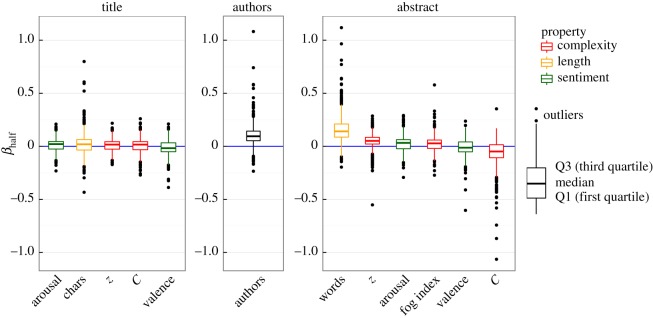
Strength of factors calculated over all journals. Box-plots (see definition on the right) summarize the distribution of *β*_half_ values across different journals. Influential factors are identified as those for which ∣β∣ is large for almost all journals (e.g. when the box does not contain *β*_half_=0 line this implies that in at least 75% of the journals the value of *β*_half_ is above or below zero).

In order to compare the strength of the effect of a factor on the number of citations, we focus on the distribution of *β*_half_ (typical papers) across different journals. The linear relationship Y=ln⁡(citations)=α+βX and the fact that *X* is standardized imply that *β* quantifies how much growth in citations should be expected from the variation of 1 standard deviation in one factor (e.g. β=ln⁡2≈0.69 means that the number of citations *Y* doubles by moving 1 standard deviation in *X*). Figure 2 summarizes the results and presents the factors ordered according to the median of the *β*_half_ distributions. The influence of factors is overall rather weak, as seen by the fact that for most journals ∣β∣<0.5. Factors in the title are considerably weaker than those in the abstract or the number of authors. The variation across journals is, in general, high, but higher in the title than in the abstract (possibly owing to the fact that the estimations of *X* are more robust in the abstract owing to the larger amount of text). The strongest factors observed are (i) the number of words in the abstract, (ii) the number of authors, and (iii) *z*-index in the abstract. For those factors, over 75% of journals (equivalently, the whole box) are placed above zero. The negative value of Herdan's *C* can be attributed to its anticorrelation to the number of words (see §4.2); when *C* is responsible for that fact and presented in the form of *z*-index the value is positive. This means that for a typical paper and for most journals a more variable vocabulary (more unique words) translates into more citations. Similarly, the number of words in the abstract or the number of authors are positively correlated with the number of citations in almost all journals.

### 2.3 Quantile dependence

Now, we quantify the extent to which the influence of factors (*β*) varies across papers with different number of citations (the quantile *τ*). We are particularly interested in the cases in which the effect of a given factor on the most successful papers is significantly different from the effect on typical papers. To quantify how typical this is, we count the number of journals for which *β*_top_≠*β*_half_ is observed beyond the estimated uncertainties *σ*_*β*_top__, *σ*_*β*_half__, i.e. $|\beta_{\mathrm{top}}-\beta_{\mathrm{half}}|>(\sigma_{\beta_{\mathrm{top}}}+\sigma_{\beta_{\mathrm{half}}})$. The results shown in table 2 reveal that overall this happens in about one-third of the cases (it is more typical for text length and less typical for sentiment factors). Table 2 also reveals the factors for which *β*_top_≠*β*_half_, because *β*(*τ*) grows in most journals (and thus *β*_top_>*β*_half_, as in the case of valence in the abstract), decays in most journals (and thus *β*_top_<*β*_half_, as in the case title length), or shows a mixed behaviour in different journals (as in the case of arousal).

**Table 2.** Factors often affect top and typical papers differently. Percentage of journals for which $\beta_{\mathrm{top}}≷\beta_{\mathrm{half}}$ are reported. The right column, *β*_top_≠*β*_half_, is the sum of the two others.

property	factor	*β*_top_>*β*_half_ (%)	*β*_top_<*β*_half_ (%)	*β*_top_≠*β*_half_ (%)
length	no. characters (title)	2.6	44.4	47.0
	no. words (abstract)	8.3	29.4	36.7
			mean	41.9
complexity	Herdan's *C* (title)	18.7	8.5	27.2
	Herdan's *C* (abstract)	34.9	6.5	41.4
	*z*-index (title)	8.3	16.7	25.0
	*z*-index (abstract)	24.6	7.7	32.3
	fog index (abstract)	26.4	8.0	34.4
			mean	32.0
sentiment	arousal (title)	11.0	13.5	24.5
	arousal (abstract)	15.7	13.7	29.4
	valence (title)	16.1	11.3	27.4
	valence (abstract)	29.2	5.7	34.9
			mean	29.1
	no. authors	4.0	39.6	43.6
			overall mean	33.7

The next question we investigate is the extent to which the quantile dependence leads to a reversal of the effect of factors, i.e. when *β*(*τ*) crosses 0. Table 3 shows the percentage of journals with positive *β*_low_, *β*_half_ and *β*_top_ coefficients for each factor. It shows that except for singular cases (marked by asterisk) the observations tend to be significantly different from chance (50%). The variation across the different *β*s (quantiles) quantifies the number of journals for which *β*(*τ*) crosses 0. Such a behaviour has already been discussed for title length in *Science* (figure 1), and table 3 confirms the generality of this observation (it shows for title length 72% of journals with positive *β*_low_ when compared with nearly 75% with negative *β*_top_). In case of three factors (title length, Herdan's *C* in the abstract, and valence in the abstract), we observe that moving from *β*_low_ to *β*_top_, we cross 50%, which indicates that for a certain range of *β* the factor in question increases the citations for most journals, whereas for other *β*s, the opposite effect is typical across journals.

**Table 3.** Percentage of journals with positive *β*_low_, *β*_half_ and *β*_top_ for each factor. All values are statistically significant (*p*<0.001) except for those marked with an asterisk (see §4.5).

property	factor	*β*_low_>0 (%)	*β*_half_>0 (%)	*β*_top_>0 (%)
length	no. characters (title)	71.4	56.2	27.7
	no. words (abstract)	96.5	96.7	83.4
complexity	Herdan's *C* (title)	50.1*	56.7	62.4
	Herdan's *C* (abstract)	19.4	28.1	51.2*
	*z*-index (title)	62.0	58.2	47.9*
	*z*-index (abstract)	71.3	82.1	81.0
	fog index (abstract)	62.9	68.2	72.9
sentiment	arousal (title)	56.5	61.6	58.3
	arousal (abstract)	62.8	67.8	61.9
	valence (title)	42.9	43.1	49.5*
	valence (abstract)	42.0	47.6*	63.4
	no. authors	93.4	92.5	61.6

The combination of the results of these two tables allows for a more complete picture of the *τ* dependence on *β* for different factors. For instance, the number of authors and the number of characters in the title can be identified as the ones that exhibit the strongest systematic trend of decaying *β*(*τ*) (in about 40% of journals, as shown in table 2). However, only for the number of authors the majority of the values are above zero (table 3), i.e. the value of *β* for top papers is less than for typical ones but it still stays positive. On the other hand, in the case of the number of characters not only is *β* smaller for top papers when compared with typical ones, but it also changes its sign. Sentiment factors (except for valence in the abstract) bring no overall information about the trend—the number of up- and downward occurrences is similar. Notably, there is a strong coincidence between *z*-index and fog index in the abstract, suggesting that although those two quantities have different definitions, both indicate the increase of correlations between abstract complexity and citations.

### 2.4 Variability across journals

**Figure 3.**
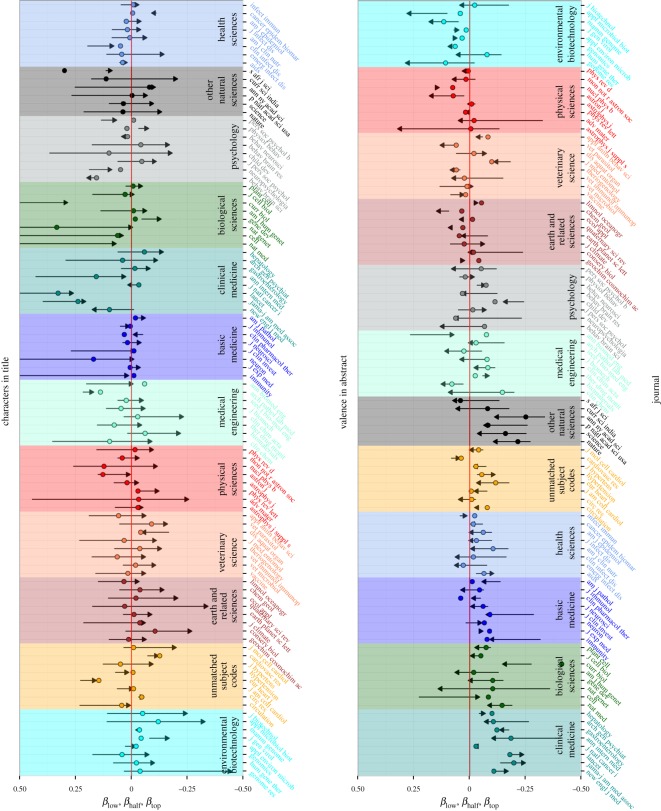
Summary pointers show *β*_low_, *β*_half_ and *β*_top_ for two factors: number of title characters (top) and valence in abstract (bottom) (see figure 1 for the definition of summary pointers). Journals are grouped according to the OECD bibliographic categories (see footnote 3). The eight journals with highest IF in each category are shown (six for *other natural sciences*). The categories are sorted with respect to the number of positive *β*_half_ values. Testing null hypothesis that categories are randomly attributed to journals (we compare the average standard deviation within categories with a random attribution of categories to journals) yields *p*-values *p*=0.002 for title length and *p*<10^−8^ for valence in abstract. The same procedure performed for IF (by creating 12 categories according to decreasing IF) gives *p*=0.02 for title length and *p*<10^−5^ for valence in abstract, suggesting higher impact exerted by scientific category.

The large variability across journals apparent in all our analysis can have different origins. One possibility is that certain journals are read only by specific (scientific) communities. To address that issue, in figure 3, we group the journals in disciplines according to their OECD subcategory^3^ and show summary pointers (introduced in figure 1) for two factors. The results indicate that the variation across journals is partially explained by disciplines, e.g. for *clinical medicine* all values of *β* in the case of valence in abstract are below zero, whereas for *physical sciences*, the majority is positive. Another possibility is that more popular journals are different from less popular journals. To address this option, journals inside each discipline in figure 3 are ranked by their IF index. No clear tendency can be visually identified; however, by comparing with a random attribution of the IF, popularity proves to be statistically significant, although to much less extent than scientific discipline (see caption for figure 3). Figure 3 also allows for a straightforward comparison of the strength of title length and abstract valence factors in different journals. By calculating exp⁡(βΔX), one can directly estimate how much gain in citations is obtained on average by a move in Δ*X* standard deviations in the variable *X* (e.g. for title length in the journal *Lancet* *β*_half_=0.33 and thus extending the length of the title by 1 standard deviation gives almost 40% gain in citations; for *Nature*, *β*_half_=0.038 and thus one obtains less than 4% gain).

## 3. Discussion and conclusion

In this paper, we investigate the *importance* of factors of scientific papers on the popularity they acquire. As factors, we consider the number of authors of the paper and text-related properties that also quantify the length of title and abstract, the complexity of the vocabulary, and sentiment based on the used words. These factors capture different stylistic dimensions of scientific writing and were also selected based on previous works that indicated a correlation to the number of citations. We found that the factors with a stronger (positive) effect on citations are the number of authors and the length of the abstract. Text complexity is positively correlated with citation at the level of the abstract, while we could not detect a strong effect within the title. The agreement of two factors designed to quantify text complexity—the *z*-index and Gunning fog index—support this conclusion (the opposite result is obtained if Herdan's *C* measure is used, but we attribute this to the negative correlation of this measure with text length). In terms of the sentiment factors, the level of arousal a title or abstract invokes is poorly correlated with citations. This result should be examined more carefully as there are controversies as to the relation between text polarity and information contained therein (see [27,28] and the following discussion). In addition, the vocabulary on which we rely in this study [29] has been obtained by evaluating the common reception of words. This fact can strongly affect the value of valence, e.g. a highly negative word ‘cancer’ in medical papers.

The discussion above, and the fact that a statistically significant effect is present for most factors, should not hide that the effect is typically weak (|*β*|<0.5 for most factors, quantiles *τ* and journals) and that there are strong fluctuations across papers and journals. For instance, a positive correlation between number of characters and citations for *all* the quantiles is measured in the *New England Journal of Medicine*, whereas a negative correlation is observed in the overwhelming majority of other journals. One of the main findings of our paper is that the factors vary also strongly depending whether the analysis uses all or only the most cited papers. We quantified this effect by the dependence of *β* on the quantile *τ* in a quantile regression analysis. One example in which this effect is particularly strong is the role of title length in figure 1. In the public media [25,26], the message behind the finding [12] of negative correlation between text length and citations was that authors should write shorter titles to achieve more citations. While this simple message is appealing and agrees with some stylistic recommendations, our results show that for most journals this is wrong (even if one assumes that there is a causal relation behind the correlations). The negative correlation is found only in the most cited journals, for typical journals, the correlation is positive (longer titles are better). This suggests that papers with short titles show a larger variation on the number of citations and can be very well cited or very poorly cited. A similar behaviour is observed in other factors, and a significant dependence on *τ* is seen on average in one-third of the journals.

Altogether, our results indicate that textual properties of title and abstract have non-trivial effects in the processes leading to the attribution of citations. In particular, the effect varies significantly between papers with the usual number of citations and with a large number of citations. This finding is even more important considering that the number of citations across papers varies dramatically. The weak signal we detect can also be considered a sign that the quantities we measure have limited information, e.g. expressing the impact of publications by a single number (the number of citations) can be misleading and lacking information (a point that has been previously raised, e.g. in [30]). The overall estimates (calculated over a set of journals or categories) may dim the clear picture one receives while observing a specific journal. For authors interested in how to write the title and abstract of their paper, we recommend looking at the values of *β*_half_ and *β*_top_ of the different factors for the specific journals of interest (tables with all factors and more than 1500 journals can be found via the Data accessibility section).

## 4. Methods

### 4.1 Data

We obtained the data from the Web of Science service about the papers marked as ‘articles’ published in the period of 1995–2004 that fulfil the following two conditions: (i) the journal where the article has been published had to be active in all the mentioned years, and (ii) there had to be at least 1000 articles published in total in this journal in the given period. By applying this filtering, we obtained over 4 300 000 articles from over 1500 different journals containing information about the title of the paper, the number of its authors, full abstract contents and OECD category it had been classified to. Additionally, for each of the records, we also recorded the number of citations it acquired between being published and 31 December 2014. Data processing, plots and statistical analysis have been performed using R language [31].

### 4.2 Text properties

The most obvious candidates for quantitative factors that could be used to describe the paper are the number of words or the number of characters. In the case of the title, the second option has been used while in the case of abstract—the first one. Additionally, the number of authors have also been used as in a previous study it had been shown to be an important factor [13]. As it concerns the complexity of the vocabulary, a way to account for that is to measure a so-called Herdan's *C* index [32], p. 72, defined for each paper *i* as

4.1 $$C_{i}=\frac{\log N_{i}}{\log M_{i}},$$

where *M*_*i*_ stands for the text length (number of words) and *N*_*i*_ is the vocabulary size (i.e. the number of unique words) of paper *i*. To overcome methodological shortcomings of this traditional approach (e.g. no fluctuations effect included) it has recently been proposed [33] to use a *z*-score that shows how much the obtained pair (*N*_*i*_, *M*_*i*_) is different from the expected value *μ*(*M*) in units of standard deviations *σ*(*M*)

4.2 $$z_{N_{i},M_{i}}=\frac{N_{i}-\mu (M_{i})}{\sigma (M_{i})},$$

where *μ*(*M*_*i*_) and *σ*(*M*_*i*_) were obtained empirically using all papers in our database. Finally, one might also take into account the complexity of the used words. A classical quantity to measure this effect is so-called Gunning fog index *F*_*i*_ [34], defined for each paper *i* as

4.3 $$F_{i}=0.4\left(\frac{{\mathrm{no}.\;\mathrm{words}}_{i}}{{\mathrm{no}.\;\mathrm{sentences}}_{i}}+100\frac{\;{\mathrm{no}.\;\mathrm{complexwords}}_{i}}{{\mathrm{no}.\;\mathrm{words}}_{i}}\right),$$

where a complex a word is a one that has more than two syllables.^4^ Fog index is widely used as its value can be connected to the number of formal years of education needed to understand the text at first reading. Because of the absence of sentences fog index has not been calculated in the case of title (i.e. a typical title contains only one sentence therefore *F*_*i*_ is highly correlated with the number of words).

### 4.3 Sentiment properties

In this study, the idea of a *dictionary, emotional classifier* has been used: in this approach, one takes the dictionary of words that had been tagged for valence and arousal and calculates the mean arithmetic value of all the recognized words. Thus, in the case of each paper, we have separately valence (and arousal) values for title and abstract. We have used a very recent study [29], which contains norms for almost 14 000 English words, where valence (*v*) and arousal (*a*) are given as real numbers in the scale of 1 to 9 (i.e. *v* below 5 is negative, whereas *v*>5 means positive words, low *a* values indicate low arousal, whereas high *a* is high arousal). The total valence and arousal were obtained as the average of all words in the title or abstract.

### 4.4 Standardization

In order to make comparison among different factors, each factor *x* has been separately standardized with respect to journal, i.e. for each *i*

4.4 $${\hat{x}}_{i}=\frac{x_{i}-\mu (x)}{\sigma (x)},$$

where *μ*(*x*) and *σ*(*x*) are, respectively, sample mean and variance over factor *x* in a journal *i* it belongs to.

### 4.5 Quantile regression

In the approach of quantile regression [22,23], having *k* factors (variables) *X*_*k*_ and an observable *Y* , we are able to obtain a regression line defined by coefficients *β*_*i*_(*τ*)

4.5 $$Y=\beta_{0}(\tau )+X_{1}\beta_{1}(\tau )+\cdots +X_{k}\beta_{k}(\tau )=X\beta$$

for a given quantile *τ* by solving the minimization problem

4.6 $$\hat{\beta }(\tau )=\arg\;\min_{\beta \in R^{k}}\sum_{i=1}^{n}(\rho_{\tau }(Y_{i}-X\beta )),$$

where $\rho_{\tau }(y)=|y(\tau -I_{\left(y<0\right)})|$ is called the loss function (I is indicator variable). In this study, we restrict ourselves to the case where

4.7 ln⁡Y=α(τ)+Xβ(τ),

i.e. we examine the influence of each of the factors separately. As the logarithm is an increasing function, the logarithm of the *p*th quantile is equal to the *p*th quantile of the log-transformed citation counts. For computational purposes, we used R's *quantreg* package [35].

### 4.6 Statistical analysis

We test if the number of positive values of *β*_low_, *β*_half_ and *β*_top_ is significantly different from the one obtained by chance (i.e. by randomly choosing ‘+’ or ‘−’ signs with equal probability $q=\frac{1}{2}$). This statistics follows a binomial distribution. However, as the number of samples (journals) *n* is large (*n*>1500), we simply use normal distribution *N*(*μ*,*σ*), with *μ*=*nq* and $\sigma =\sqrt{nq(q-1)}$ with $q=\frac{1}{2}$. We consider the observation to be statistically significant if the measured number of positive *β* differs from *μ* by more than 3*σ* (i.e. *p*-value is less than 0.001).

## Footnotes

1 This guarantees that papers have at least 10 years to gain citations. Equivalent, but much more noisy (owing to drastically limited number of data points), results are obtained when looking only at papers published in the same year.

2 We perform QR fitting for each journal independently, because journals are known to play an important role in the number of citations a paper receives.

3 See http://www.oecd.org/science/inno/38235147.pdf.

4 An own adaptation of Greg Fast's Pearl algorithm (http://cpansearch.perl.org/src/GREGFAST/Lingua-EN-Syllable-0.251/Syllable.pm) to R language has been used.

## Data accessibility

Tables containing: (i) exemplary information enabling to recover the top panel of figure 1 (quantile dependence for number of characters in *Science* journal in years 1995–2004) as well as (ii) aggregated information about quantile regression slope coefficients *β*: *β*_low_≡*β*(*τ*=0.02), *β*_half_≡*β*(*τ*=0.5) and *β*_top_≡*β*(*τ*=0.98) and their uncertainties *σ*_*β*_low__, *σ*_*β*_half__ and *σ*_*β*_top__ for all examined journals and factors (enabling recovery of figures 2 and 3 as well as tables 2 and 3) are available as Dryad Digital Repository http://dx.doi.org/10.5061/dryad.nj938.
